# Ultradeep, targeted sequencing reveals distinct mutations in blood compared to matched bone marrow among patients with multiple myeloma

**DOI:** 10.1038/s41408-019-0238-0

**Published:** 2019-09-30

**Authors:** David G. Coffey, Qian V. Wu, Andrea M. H. Towlerton, Sharon Ornelas, Alicia J. Morales, Yuexin Xu, Damian J. Green, Edus H. Warren

**Affiliations:** 1 Clinical Research Division, Fred Hutchinson Cancer Research Center, Seattle, WA USA; 2 Department of Medicine, Division of Medical Oncology, University of Washington, Seattle, WA USA

**Keywords:** Cancer genomics, Myeloma, Genetics research, Translational research, Cancer genomics

Dear Editor,

Multiple myeloma (MM) is an incurable plasma cell neoplasm that evolves from monoclonal gammopathy of undetermined significance (MGUS) and smoldering multiple myeloma (SMM). Primary genomic events responsible for the development of MGUS and SMM include hyperdiploidy, translocations, and copy number alterations^1^. Secondary events causing the transformation to active MM are driven by the acquisition of somatic mutations, including single-nucleotide variants (SNVs) and insertions and deletions (indels)^1^. Large-scale genomic studies, primarily of newly diagnosed MM, have identified at least 65 significant, recurrently mutated genes affecting 5 distinct cellular pathways^1,2^.

Spatial and temporal tumor heterogeneity represent major challenges to the study of MM genomics^3^. The current standard of care method for sampling MM tumor cells involves an invasive bone marrow biopsy. However, doing so may lead to overestimation or underestimation of the total disease burden, and if sequenced, its genomic profile may not be reflective of the entirety of the tumor population. For this reason, sequencing of circulating tumor DNA (ctDNA) from blood is under evaluation as a complementary or possibly alternative method for sampling the malignant DNA^4–7^. Theoretically, ctDNA sequencing may reveal mutations from multiple, non-contiguous sites of disease, including extramedullary disease. Minimally invasive phlebotomy facilitates frequent sampling enabling detection of clonal evolution at shorter time intervals.

Sequencing myeloma tumor cells in the bone marrow or blood has traditionally been performed on sorted CD138^+^ plasma cells. However, sorting the tumor population by flow or magnetic-activated cell sorting does not guarantee enrichment of the malignant plasma cell population. This is because non-malignant plasma cells commonly express the same cell surface proteins as malignant plasma cells. Furthermore, subpopulations of malignant plasma cells may lose expression of typical cell surface proteins that could result in a systematic bias.

The aim of this study was to compare the mutational profile of genes recurrently mutated in myeloma within bulk, unsorted circulating blood and bone marrow mononuclear cells among patients with MM at diagnosis, relapse, and remission. In addition, we extensively studied the somatic mutations within the peripheral blood from a single patient with relapsed MM who underwent sampling monthly over 3.8 years. We hypothesized that somatic mutations detected in the blood may have clinical significance, regardless of their cell of origin, and may correlate with disease burden.

A total of 126 samples were sequenced: 38 paired peripheral blood mononuclear cells (PBMCs) and bone marrow mononuclear cells (BMMCs) samples obtained on the same day (Supplementary Table 1), 39 PBMC samples from a single MM patient collected approximately monthly for 3.8 years, and 11 PBMC samples from 11 healthy donors (Supplementary Table 2). The patient whom we collected serially was diagnosed with International Staging System (ISS) stage I MM 7.3 years prior to enrollment and had previously progressed on 9 different lines of therapy. For the duration of monitoring, the patient was treated with single-agent isatuximab (Sanofi Genzyme, Cambridge, MA, USA) within a clinical trial.

Using a custom 49-gene panel of significant, recurrently mutated genes in myeloma (Supplementary Table 3), we sequenced to a mean depth of 4143× using a next-generation sequencing library that incorporated unique molecular identifiers (see Supplementary Methods). Somatic mutations were detected in PBMCs in 90% of MM patients compared to 18% in healthy individuals (Fisher’s exact test, *p* = 1.582 × 10^−5^). The mean number of somatic variants was 12 per person in MM patients compared to 0.3 in healthy individuals (Wilcoxon rank-sum test, *p* = 2.641 × 10^−5^). The mean variant allele frequency (VAF) was 5.8% in MM patients compared to 1.6% in healthy individuals (Wilcoxon rank-sum test, *p* = 0.039). Genes mutated in the healthy individuals included *TP53* (*n* = 1), *TRAF3* (*n* = 1), and *MAX* (*n* = 1).

Among the 38 paired blood and bone marrow samples, 6164 SNVs or indels were detected, of which 503 met our definition of somatic variants (Supplementary Fig. 1). The average VAF was 5.8% in PBMCs and 4.0% in BMMCs (Supplementary Fig. 2). Among the 503 somatic variants detected in the blood and marrow (Supplementary Fig. 3), only 3% of somatic variants were shared by both tissue compartments within the same individual and the majority were in the gene *DNMT3A* (Fig. 1a). Among the 13 shared variants, a positive, linear correlation in VAF was detected (Pearson’s *R* = 0.95).

**Fig. 1.**
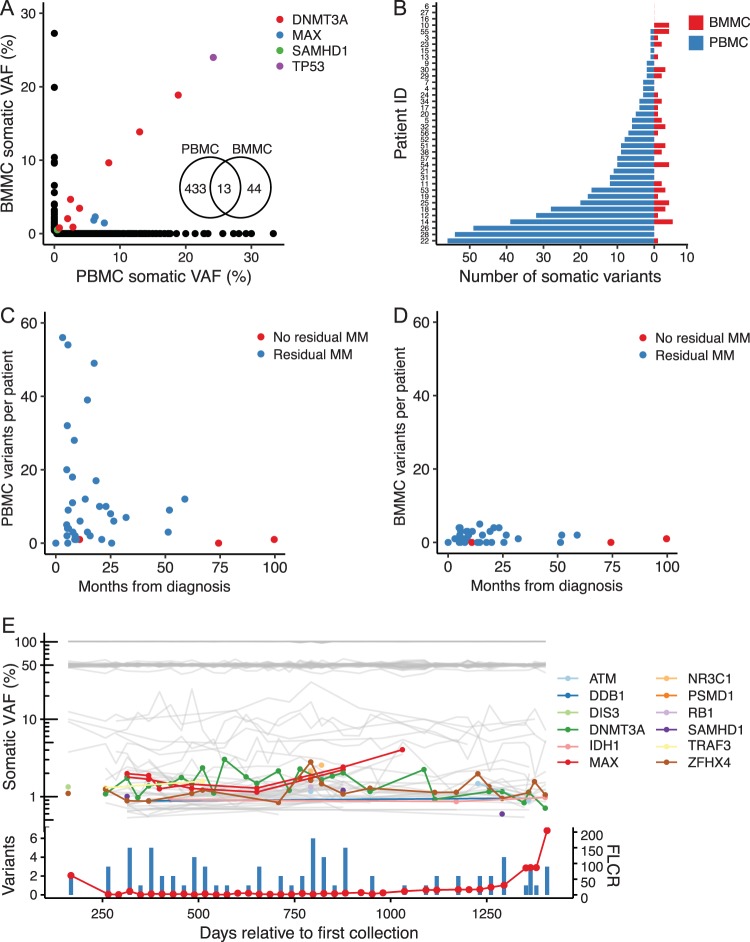
Somatic mutations detected in unsorted PBMC and BMMC samples from patients with MM. **a** Scatter plot of somatic mutation VAF detected in paired PBMC and BMMC samples. **b** Number of somatic variants detected per patient by tissue type. **c** Number of somatic variants relative to months from diagnosis in PBMC and **d** BMMC samples. Indicated in blue are samples with measurable disease, defined by the presence of an abnormal free light chain ratio, elevated M-protein, circulating plasma cells, abnormal bone marrow plasma cells, or bony lesions on X-ray, CT, or MRI. **e** VAF (top) and total number of coding somatic variants (bottom, blue bars) detected in serially acquired PBMC samples from a single patient with relapsed multiple myeloma collected approximately monthly for 3.8 years. Gray lines in the top panel indicate VAF of germline and non-coding variants. Bottom, red line shows the serum free light chain ratio (FLCR) over the period of time blood was sampled

The mean number of variants per patient was 12 in the blood compared to 2 in the bone marrow (Wilcoxon signed-rank test, *p* = 8.806 × 10^−6^, Fig. 1b). Missense SNVs, particularly transversions, accounted for the largest difference between PBMC and BMMC samples (Supplementary Fig. 4). The number of somatic variants in PBMCs was significantly higher among MM patients with any measurable disease and among patients who had detectable bony lytic lesions (Table 1). The number of somatic variants in PBMCs was higher within the first 2 years of diagnosis but sharply declined thereafter (Fig. 1c, d). There was no significant difference in the number of mutations between PBMCs and BMMCs among patients in remission without detectable disease (Wilcoxon rank-sum test, *p* = 0.619). There was also no significant difference in the number of detectable mutations and exposure to prior classes of drug therapy for PBMCs (analysis of variance *p* = 0.986) or BMMCs (*p* = 0.552).

**Table 1:** Mean number of somatic variants per sample by clinical variables

	PBMCs				BMMCs			
	Mean	SD	*N*	*p*	Mean	SD	*N*	*p*
Any measurable disease								
Absent	0.67	0.58	3	**0.024**	0.33	0.58	3	0.146
Present	12.68	15.5	35		1.60	0.58	35	
Bony lytic lesion								
Absent	3.71	6.37	7	**0.036**	1.14	1.46	7	0.425
Present	13.60	16.40	30		1.63	1.50	30	
M-protein or free light chain ratio								
Normal	9.57	12.80	23	0.098	1.41	1.33	22	0.451
Abnormal	18.50	19.10	12		1.92	1.75	13	
Bone marrow plasma cells								
<10%	11.10	15.00	27	0.135	1.57	1.55	28	0.651
≥10%	16.90	18.10	8		1.25	1.49	8	
Circulating plasma cells								
Absent	3.00	5.20	3	0.241	0.67	1.15	3	0.185
Present	13.70	18.70	6		2.33	1.63	6	
Risk stratification								
High risk	10.70	14.10	14	0.320	1.64	1.65	14	0.160
Standard risk	9.75	18.50	8		0.63	0.92	8	
Age								
< 50 years	13.10	17.40	8	0.641	1.12	1.36	8	0.471
≥ 50 years	11.40	14.90	30		1.60	1.52	30	
Transplant status								
Pretransplant	12.80	16.90	24	0.880	1.42	1.47	24	0.696
Posttransplant	9.86	12.20	14		1.64	1.55	14	

*PBMC* peripheral blood mononuclear cells; *BMMC* bone marrow mononuclear cells; Mean, mean number of somatic variants per sample; *SD* standard deviation; *N* number of patients; p, Wilcoxon rank-sum test *p* value. For comparisons where the *N* < 5, caution is needed for interpretation due to small sample size

The 5 most frequently mutated genes across the 38 patients with paired blood and marrow samples were *DNMT3A* (33%), *MAX* (30%), *ATM* (26%), *SAMHD1* (22%), and *FGFR3* (18%) (Supplementary Fig. 5). Genes that have been implicated in clonal hematopoiesis of indeterminate potential (CHIP) (*DNMT3A*, *ATM*, *TP53*)^8^ were among the most frequently mutated, and mutations in one or more of these genes were detected in 30 of the 38 MM patients (79%). There was no significant association between the detection of a CHIP mutation and age (Wilcoxon rank-sum test, *p* = 0.507) or transplant status (Fisher’s exact test, *p* = 0.628). The most common amino acid changes affecting ≥10 samples were in *MAX* (S78fs, S79fs), *FGFR3* (L309H), *ATM* (D273fs), and *SAMHD1* (F329L) (Supplementary Fig. 6). The genes *ATM*, *FGFR3*, *SAMHD1*, *MAX*, and *NCKAP5* were found to be significantly, differentially mutated in PBMC compared to BMMC samples (Supplementary Fig. 7).

In the MM patient with PBMC samples serially acquired over 3.8 years, there were 21 somatic mutations detected at multiple time points involving 12 genes. The VAF of these mutations fluctuated over time (range, 0.6–4%) but did not appear to change in frequency at the time of relapse nor did the total number of mutations (Fig. 1e).

To our knowledge, this is the first report comparing ultradeep sequencing of synchronously acquired, unsorted PBMCs and BMMCs in patients with MM. Our three major findings are: (1) significantly greater number of somatic mutations was detected in PBMCs compared to BMMCs; (2) the number of somatic mutations in PBMCs was greater for patients with elevated markers for disease, especially bony lytic lesions; (3) genes implicated in CHIP were among the most commonly mutated genes (*DNMT3A*, *ATM*, *TP53*).

These findings have significant relevance to the field of ctDNA. Most notably, the observation that PBMCs are a richer source of somatic mutations suggests that sequencing the blood may reveal mutations not detectable from a bone marrow biopsy. One possible explanation for this finding may be that circulating blood comes into contact and mixes with multiple sites of disease such that a single blood sample is more representative of the spatial genomic heterogeneity than is a single bone marrow sample. This hypothesis is supported by our finding that patients with bony lytic lesions have more than three times as many circulating somatic mutations than patients whose disease is not detectable by imaging.

Although the clinical significance of these somatic mutations is uncertain and their cell of origin is not known, two observations suggest that they are disease or treatment related. First, we observed significantly more somatic mutations in the blood from patients with MM than in healthy controls. Second, among patients with MM, the number of mutations detected was significantly higher in patients with measurable disease. These findings would suggest that measuring circulating somatic mutations may have prognostic or potentially predictive value.

The finding that more somatic mutations in PBMCs were detected within the first 2 years of diagnosis followed by a decline thereafter was intriguing (Fig. 1c). One potential explanation for this observation is that patients who are less likely to live beyond the first 2 years are more likely to have more rapidly progressive disease resulting in higher ctDNA production. This is supported by the observation that there were a disproportionately higher number of patients with ISS stage III disease (44%) who were sampled in the first 2 years compared to patients sampled at later time points (14%). It may be for this reason that we detected a low level of mutations in the patient whom we studied serially as he had been living with his diagnosis for 10 years. In contrast, patients who have more aggressive disease and die within the first 2 years of diagnosis may be more likely to have a greater mutational burden resulting in frequent treatment resistance and early mortality.

Our observation that genes implicated in CHIP were among the most commonly mutated genes was especially interesting. CHIP is defined as the detection of somatic mutations at a VAF > 2% in genes recurrently mutated in hematologic malignancies in the absence of morphologic evidence of a hematological neoplasm^8^. Our assays did not include all previously reported CHIP genes such as *TET2* and *ASXL1* since they were not previously reported as frequently mutated in myeloma. Analogous to MGUS, CHIP mutations have been associated with progression to myeloid neoplasms at a rate of 0.5–1% per year^8^. However, to our knowledge, CHIP mutations have not been associated with progression to plasma cell neoplasms. Although we did not identify an association between the development of secondary malignancies and the presence of CHIP mutations, our study was not powered to detect such an association and further investigation is warranted.

## Supplementary information

Supplemental information
