# Supplementary material for: Ultradeep, targeted sequencing reveals distinct mutations in blood compared to matched bone marrow among patients with multiple myeloma
Source: Blood Cancer J. 2019 Sep 30;9(10):77. doi: 10.1038/s41408-019-0238-0 (PMC6768874; doi:10.1038/s41408-019-0238-0)
Supplement: Supplementary file 1 — Supplemental information [file 41408_2019_238_MOESM1_ESM.docx]

Ultradeep, targeted sequencing reveals distinct mutations in blood compared to matched bone marrow among patients with multiple myeloma

**Supplementary Information**

David G. Coffey^1,2^

Qian V. Wu^1^

Andrea M.H. Towlerton^1^

Sharon Ornelas^1^

Alicia J. Morales^1^

Yuexin Xu^1^

Damian J. Green^1,2^

Edus H. Warren^1,2^

^1^Clinical Research Division, Fred Hutchinson Cancer Research Center, Seattle, WA

^2^Department of Medicine, Division of Medical Oncology, University of Washington, Seattle, WA

SUPPLEMENTARY METHODS

**Sample preparation**

Informed consent was obtained from all patients and healthy donors in accordance with protocols approved by the Institutional Review Board at the Fred Hutchinson Cancer Research Center. Blood and marrow were collected in sodium citrate or EDTA vacutainers. Peripheral blood mononuclear cells (PBMC) and bone marrow mononuclear cells (BMMC) were isolated by ficoll density gradient centrifugation, cryopreserved in DMSO or CyroStor (BioLifeSolutions, Bothell, WA, USA), and stored in liquid nitrogen. DNA was extracted from mononuclear cells using the QIAmp Blood Mini Kit (QIAGEN, Venlo, Netherlands) or by automated purification using Autopure reagents (Autogen, Holliston, MA, USA). The purity of the DNA was assessed by a NanoDrop spectrophotometer and double-stranded DNA concentration was quantified by Qubit (ThermoFisher, Waltham, MA, USA).

**Targeted sequencing of mononuclear cells**

A custom 49-gene panel (Supplementary Table 2) of significant, recurrently mutated genes in myeloma was selected based on reported findings from an interim analysis of the CoMMpass study (NCT145429).^1^ Our panel included 3 genes which are known to be mutated in clonal hematopoiesis of indeterminate potential (CHIP): *DNMT3A*, *ATM*, and *TP53*.^2^ We chose targeted DNA sequencing over whole exome or whole genome sequencing since DNA sequencing of a smaller region of the genome allowed us to maximize depth and thereby increase sensitivity to detect rare somatic variants. Next generation sequencing libraries were prepared using the QIAseq Targeted DNA kit (QIAGEN). This method incorporates unique molecular identifiers (UMI) on individual DNA molecules so that PCR and sequencing artifacts can be readily removed *in silico*. The input DNA amount for each sample was 100 ng measured by Qubit. The fragment size of the multiplexed library was verified by TapeStation (Agilent, Santa Clara, CA, USA). Libraries meeting all quality control standards were sequenced on a HiSeq 2500 (Illumina, San Diego, CA, USA) and paired-end 150 bp reads were generated to target an average depth of 5,000X.

**Bioinformatic and statistical analysis**

Fastq files were demultiplexed, trimmed of barcode and adaptor sequences, and aligned to the human genome (Hg19) using the Burrows-Wheeler Aligner.^3^ Reads less than 40 bp, not aligning to the target region, or not passing mapping quality filters were discarded from the analysis. After removing 18% of reads that did not pass quality thresholds, the mean number of unique molecules detected per sample was 2.7 x 10^6^ and the mean number of reads per molecule was 5. This resulted in a mean read depth of 4,143X being achieved per sample.

SNVs and indels were identified using smCounter2 (<https://github.com/qiaseq/qiaseq-smcounter-v2>), a software tool that integrates UMIs into a Bayesian probabilistic model to call nucleotide variants.^4^ Previous research has demonstrated that smCounter2 can detect variants with ≥ 0.5% allele fraction in coding regions with over 90% sensitivity and less than 10 false positives per megabase.^4^ All default settings were used: minimum base quality = 25, minimum mapping quality = 50, minimum number of alternative UMIs = 3, average number of mismatches per 100 bases allowed = 6, threshold on read proportion to determine UMI level consensus = 0.8, minimum length for homopolymers = 10, filter variants that are within 2 bases to primer.

Variant calling files were annotated using ANNOVAR.^5^ After removal of low-quality variants using smCounter2 default settings, candidate somatic variants were defined as those with a populational allele frequency less than 5% reported in to the Exome Aggregation Consortium (ExAC) database^6^ and variant allele frequency (VAF) less than 37% or within the CoMMpass IA12a database (Supplementary Figure 1). The VAF threshold of 37% used to filter somatic variants was determined from the following formula:

$$Somatic VAF threshold= 0.5-\mu_{\left( P_{i}-B_{i} \right)}+2\cdot\sigma_{\left( P_{i}-B_{i} \right)}$$

where μ is the mean and 𝜎 is the standard deviation of the difference between all paired PBMC (P_i_) and BMMC (B_i_) germline VAF where the frequency is expected to be the same but differs due to technical artifact. For this calculation, a germline VAF was defined as a variant passing all quality filters and having a population allele frequency greater than 5% in the ExAC database. Additional filtering was performed to remove all synonymous, non-exonic variants since they have a low potential to be deleterious to protein function.

For continuous variables, we used non-parametric Wilcoxon test instead of two-sample t-test in the analysis, which is more robust and distribution-free. Wilcoxon signed-rank test was used to compare numerical variables between two paired groups, e.g. PBMC vs BMMC; Wilcoxon rank-sum test was used for two independent group comparison, e.g., age > 50 vs age < 50. For groups with multiple levels (e.g. classes of drug therapy), one-way analysis of variance (ANOVA) was used. For categorical variables, Fisher’s exact test was used compare categorical variables between two independent groups (PBMC vs healthy control) and McNemar’s test was used to compare between two paired groups (PBMC vs BMMC). Maftools R package was used to generate oncoplots, lollipop plots, and forest plots of differentially mutated genes.^7^

**Supplementary Table 1.** Multiple myeloma population studied for BMMC and PBMC comparison (n = 38 patients). Samples were collected between 2004 and 2015 from patients treated at the Seattle Cancer Care Alliance. X, previous therapy administered, PI, proteasome inhibitor (bortezomib, carfilzomib); IMiD, immunomodulatory drug (thalidomide, lenalidomide, pomalidomide); ASCT, melphalan-conditioned autologous stem cell transplant; ALK, alkylator (cyclophosphamide, bendamustine, cisplatin); TI, topoisomerase inhibitor; AM, anti-microtubular agent (vincristine).

| **ID** | **Age** | **Sex** | **ISS Stage** | **Status** | **Months after diagnosis** | **PI** | **IMiD** | **ASCT** | **ALK** | **TI** | **AM** | **PBMC**  **Variants** | **BMMC Variants** |
| --- | --- | --- | --- | --- | --- | --- | --- | --- | --- | --- | --- | --- | --- |
| 22 | 59 | Female | I | Relapse | 3 |  | X |  |  |  |  | 56 | 1 |
| 28 | 29 | Male | II | Remission | 6 | X | X |  |  |  |  | 54 | 0 |
| 26 | 51 | Male | III | Relapse | 18 | X | X | X | X | X |  | 49 | 0 |
| 14 | 58 | Male |  | Relapse | 14 |  | X |  |  | X | X | 39 | 5 |
| 12 | 57 | Male | III | Relapse | 5 | X |  |  |  | X |  | 32 | 1 |
| 18 | 55 | Male | I | Relapse | 8 |  | X |  |  |  |  | 28 | 3 |
| 25 | 62 | Male | II | Relapse | 5 |  | X |  |  |  |  | 20 | 4 |
| 53 | 57 | Male |  | Relapse | 19 |  |  | X | X | X | X | 17 | 3 |
| 19 | 45 | Male | I | Relapse | 8 |  | X |  |  |  |  | 18 | 1 |
| 11 | 58 | Male | I | Relapse | 59 | X | X |  |  | X |  | 12 | 2 |
| 54 | 57 | Male |  | Relapse | 23 | X |  | X | X | X | X | 10 | 4 |
| 31 | 61 | Female | I | Relapse | 13 |  | X | X | X | X |  | 12 | 0 |
| 51 | 57 | Female | II | Remission | 6 |  | X | X | X | X | X | 9 | 3 |
| 21 | 50 | Female | III | Relapse | 8 |  | X |  |  | X | X | 11 | 0 |
| 38 | 65 | Male | I | Remission | 52 | X | X |  |  | X | X | 9 | 2 |
| 57 | 56 | Male | III | Relapse | 20 |  |  | X | X | X |  | 10 | 0 |
| 32 | 56 | Male | III | Relapse | 11 |  | X | X |  |  |  | 6 | 3 |
| 5 | 68 | Male | II | Remission | 26 |  | X | X | X | X | X | 6 | 2 |
| 52 | 38 | Male | III | Remission | 25 |  | X |  |  | X | X | 8 | 0 |
| 56 | 47 | Female |  | Relapse | 32 | X | X | X | X | X |  | 7 | 1 |
| 20 | 48 | Male | II | Relapse | 5 |  | X |  |  |  |  | 5 | 1 |
| 34 | 58 | Male | II | Relapse | 5 |  | X |  |  |  |  | 4 | 2 |
| 17 | 61 | Female | III | Relapse | 6 |  | X |  |  |  |  | 4 | 1 |
| 30 | 65 | Female | III | Remission | 9 |  | X |  |  |  |  | 2 | 3 |
| 55 | 55 | Male | III | Remission | 21 |  | X | X | X | X |  | 1 | 4 |
| 10 | 48 | Female | III | Remission | 5 |  |  |  |  | X | X | 0 | 4 |
| 24 | 57 | Male | III | Remission | 8 | X | X |  |  | X |  | 3 | 1 |
| 29 | 47 | Male |  | Relapse | 16 | X |  | X | X | X |  | 2 | 2 |
| 4 | 74 | Male | II | Relapse | 51 | X | X |  | X | X |  | 3 | 0 |
| 7 | 60 | Female | I | Relapse | 14 | X | X |  |  |  |  | 3 | 0 |
| 23 | 67 | Female | III | Relapse | 9 | X |  |  |  | X |  | 1 | 2 |
| 3 | 60 | Male |  | Remission | 100 | X | X | X | X | X | X | 1 | 1 |
| 9 | 74 | Female | II | Relapse | 5 |  | X |  |  |  |  | 2 | 0 |
| 13 | 58 | Female | II | Relapse | 9 | X | X |  |  |  |  | 1 | 1 |
| 15 | 61 | Male | II | Remission | 11 |  | X |  |  |  |  | 1 | 0 |
| 6 | 60 | Male | I | Pre-treatment | 0 |  |  |  |  |  |  | 0 | 0 |
| 16 | 50 | Female | I | Relapse | 26 |  | X |  |  |  |  | 0 | 0 |
| 27 | 66 | Female |  | Remission | 74 | X | X | X | X | X | X | 0 | 0 |

**Supplementary Table 2.** Characteristics of the healthy donor population (n = 11 individuals). Individuals with a history of a hematologic disorder or who have a significantly abnormal complete blood count were excluded from donation.

| **ID** | **Age** | **Sex** | **PBMC Variants** |
| --- | --- | --- | --- |
| 40 | 61 | Male | 0 |
| 48 | 33 | Male | 0 |
| 41 | 49 | Male | 0 |
| 42 | 46 | Male | 0 |
| 43 | 52 | Female | 1 |
| 49 | 45 | Male | 0 |
| 44 | 36 | Male | 0 |
| 45 | 35 | Male | 0 |
| 46 | 44 | Male | 0 |
| 47 | 64 | Female | 2 |
| 50 | 37 | Male | 0 |

**Supplementary Table 3.** Target gene panel (n = 49 genes).

| **Symbol** | **Name** | **Chromosome** | **Position** | **CoMMpas IA12a prevalence (%)** |
| --- | --- | --- | --- | --- |
| *KRAS* | Kirsten rat sarcoma viral oncogene homolog | 12 | p12.1 | 30.1 |
| *NRAS* | neuroblastoma RAS viral (v-ras) oncogene homolog | 1 | p13.2 | 26.0 |
| *FAM46C* | family with sequence similarity 46, member C | 1 | p12 | 12.6 |
| *DIS3* | DIS3 mitotic control homolog (S. cerevisiae) | 13 | q22.1 | 11.7 |
| *TRAF3* | TNF receptor-associated factor 3 | 14 | q32.32 | 9.3 |
| *BRAF* | v-raf murine sarcoma viral oncogene homolog B | 7 | q34 | 8.4 |
| *TP53* | tumor protein p53 | 17 | p13.1 | 7.3 |
| *EGR1* | early growth response 1 | 5 | q31.2 | 5.5 |
| *HIST1H1E* | histone cluster 1, H1e | 6 | p22.2 | 4.9 |
| *ATM* | ataxia telangiectasia mutated | 11 | q22.3 | 4.7 |
| *ZFHX4* | zinc finger homeobox 4 | 8 | q21.11 | 4.5 |
| *FGFR3* | fibroblast growth factor receptor 3 | 4 | p16.3 | 4.1 |
| *MAX* | MYC associated factor X | 14 | q23.3 | 4.1 |
| *PRKD2* | protein kinase D2 | 19 | q13.32 | 3.9 |
| *ACTG1* | actin, gamma 1 | 17 | q25.3 | 3.8 |
| *SP140* | SP140 nuclear body protein | 2 | q37.1 | 3.8 |
| *CYLD* | cylindromatosis (turban tumor syndrome) | 16 | q12.1 | 3.5 |
| *LTB* | lymphotoxin beta (TNF superfamily, member 3) | 6 | p21.33 | 3.5 |
| CCND1 | cyclin D1 | 11 | q13.3 | 3.2 |
| *IRF4* | interferon regulatory factor 4 | 6 | p25.3 | 3.0 |
| *FUBP1* | far upstream element (FUSE) binding protein 1 | 1 | p31.1 | 2.9 |
| *PRDM1* | PR domain containing 1, with ZNF domain | 6 | q21 | 2.9 |
| *NCKAP5* | NCK-associated protein 5 | 2 | q21.2 | 2.8 |
| *SAMHD1* | SAM domain and HD domain 1 | 20 | q11.23 | 2.8 |
| *PTPN11* | protein tyrosine phosphatase, non-receptor type 11 | 12 | q24.13 | 2.4 |
| *RB1* | retinoblastoma 1 | 13 | q14.2 | 2.4 |
| *NFKBIA* | nuclear factor of kappa light polypeptide gene enhancer in B-cells inhibitor, alpha | 14 | q13.2 | 2.2 |
| *BCL7A* | B-cell CLL/lymphoma 7A | 12 | q24.31 | 2.1 |
| *TRAF2* | TNF receptor-associated factor 2 | 9 | q34.3 | 2.0 |
| *RASA2* | RAS p21 protein activator 2 | 3 | q23 | 1.9 |
| *TGDS* | TDP-glucose 4,6-dehydratase | 13 | q32.1 | 1.7 |
| *XBP1* | X-box binding protein 1 | 22 | q12.1 | 1.3 |
| *ATR* | ataxia telangiectasia and Rad3 related | 3 | q23 | 1.1 |
| DNMT3A | DNA (cytosine-5-)-methyltransferase 3 alpha | 2 | p23.3 | 0.9 |
| *FCF1* | FCF1 rRNA-processing protein | 14 | q24.3 | 0.8 |
| *IKZF3* | IKAROS family zinc finger 3 (Aiolos) | 17 | q12 | 0.8 |
| *STAT3* | signal transducer and activator of transcription 3 (acute-phase response factor) | 17 | q21.2 | 0.8 |
| *ATRIP* | ATR interacting protein | 3 | p21.31 | 0.6 |
| *CUL4B* | cullin 4B | X | q24 | 0.6 |
| *DDB1* | damage-specific DNA binding protein 1, 127kDa | 11 | q12.2 | 0.6 |
| *CRBN* | cereblon | 3 | p26.2 | 0.5 |
| IDH1 | isocitrate dehydrogenase 1 (NADP+), soluble | 2 | q34 | 0.5 |
| *PSMD1* | proteasome (prosome, macropain) 26S subunit, non-ATPase, 1 | 2 | q37.1 | 0.5 |
| *CUL4A* | cullin 4A | 13 | q34 | 0.3 |
| IDH2 | isocitrate dehydrogenase 2 (NADP+), mitochondrial | 15 | q26.1 | 0.3 |
| *NR3C1* | nuclear receptor subfamily 3, group C, member 1 (glucocorticoid receptor) | 5 | q31.3 | 0.3 |
| PIK3CA | phosphatidylinositol-4,5-bisphosphate 3-kinase, catalytic subunit alpha | 3 | q26.32 | 0.2 |
| *IKZF1* | IKAROS family zinc finger 1 (Ikaros) | 7 | p12.2 | 0.1 |
| *PSMB8* | proteasome (prosome, macropain) subunit, beta type, 8 | 6 | p21.32 | 0.1 |

**Supplementary Figure 1.** Somatic variant filtering scheme and number of detected variants with each filter for paired BM and PBMC samples (n = 76 samples).

**Supplementary Figure 2.** Variant allele frequencies for all putative somatic and germline variants detected in 49 target gene panel across BM and PBMC samples collected from 38 patients with multiple myeloma and 11 healthy donors PBMC samples.

**Supplementary Figure 3**. Frequency of non-synonymous somatic mutations in 49 genes recurrently mutated in MM that were detected in synchronously acquired, paired BMMC and PBMC samples from 38 MM patients. One or more gene alterations were detected in 59 of 76 samples (78%). At least one mutation was detected in all 49 genes sequenced. Twenty-four genes (55%) were mutated in both BMMC and PBMC samples. Symbol color indicates the class of mutation, and grey indicates that no somatic mutation was detected.

**Supplementary Figure 4**. A) SNV classification, B-C) variant type in paired BMMC and PBMC samples. Error bars represent standard error of the mean. Ti, transition; Tv, transversion; C, cytosine; A, adenine; T, thymine; G, guanine; DEL, deletion; INS, insertion; SUB, substitution; SNP, single nucleotide variant; FS, frameshift; IF, in-frame; MSM, missense mutation; NSM, non-sense mutation.

**Supplementary Figure 5.** Oncoplot comparing the 10 most frequently mutated genes in BMMC and PBMC samples.

**Supplementary Figure 6.** Lollipop plots showing the number of samples and location of amino acid changes in genes from PBMC (above gene model) and BMMC samples (below gene model). Only genes in which 10 or more patients were found to have the same mutation are shown.

**Supplementary Figure 7.** Significant, differentially mutated genes in PBMC and BMMC samples determined by McNemar’s test. Benjamini and Hochberg false discovery rate (FDR) adjusted p-value, *** < 0.001, ** < 0.01, * < 0.05.

REFERENCES

1 Keats J. J. et al. Molecular Predictors of Outcome and Drug Response in Multiple Myeloma: An Interim Analysis of the Mmrf CoMMpass Study. *Blood* 2016; **128**: 194.

2 Steensma D. P. et al*.* Clonal hematopoiesis of indeterminate potential and its distinction from myelodysplastic syndromes. *Blood* 2015; **126**: 9–16.

3 Li H., Durbin R. Fast and accurate short read alignment with Burrows-Wheeler transform. *Bioinformatics* 2009; **25**: 1754 1760.

4 Xu C. et al. smCounter2: an accurate low-frequency variant caller for targeted sequencing data with unique molecular identifiers. *Bioinformatics* 2018; : 1–11.

5 Yang H., Wang K. Genomic variant annotation and prioritization with ANNOVAR and wANNOVAR. *Nat Protoc* 2015; **10**: 1556 1566.

6 Lek M. et al. Analysis of protein-coding genetic variation in 60,706 humans. *Nature* 2016; **536**: 285–291.

7 Mayakonda A., Lin D-C, Assenov Y., Plass C., Koeffler P. H. Maftools: efficient and comprehensive analysis of somatic variants in cancer. *Genome Res* 2018; **28**: 1747–1756.
